# Optimizing clinical monitoring and management guidelines for capivasertib in HR-positive/HER2-negative advanced breast cancer: expert opinion

**DOI:** 10.1038/s41523-025-00864-2

**Published:** 2025-12-04

**Authors:** Neil M. Iyengar, Joyce A. O’Shaughnessy, Heather N. Moore, Erika Hamilton, Komal L. Jhaveri, Cynthia X. Ma, Karen Tedesco, Monali Vasekar, Sarah Donahue, Hope S. Rugo

**Affiliations:** 1 Winship Cancer Institute of Emory University, Atlanta, GA USA; 2 Texas Oncology, Baylor University Medical Center, US Oncology, Dallas, TX USA; 3 Duke University Health System, Durham, NC USA; 4 Sarah Cannon Research Institute, Nashville, TN USA; 5 Tennessee Oncology, Nashville, TN USA; 6 Department of Medicine, Memorial Sloan Kettering Cancer Center, New York, NY USA; 7 Department of Medicine, Weill Cornell Medical College, New York, NY USA; 8 Washington University School of Medicine, St Louis, MO USA; 9 New York Oncology Hematology, US Oncology Network, Albany, NY USA; 10 Pennsylvania State University College of Medicine, Hershey, PA USA; 11 San Francisco Helen Diller Family Comprehensive Cancer Center, University of California, San Francisco, CA USA; 12 City of Hope Comprehensive Cancer Center, Duarte, CA USA

**Keywords:** Cancer, Diseases, Oncology

## Abstract

Capivasertib, an AKT inhibitor, approved in combination with fulvestrant for hormone receptor–positive, human epidermal growth factor receptor 2–negative advanced breast cancer with ≥1 *PIK3CA*, *AKT1*, and/or *PTEN* alterations, significantly improved progression-free survival in the CAPItello-291 phase 3 trial. However, capivasertib-associated adverse events of diarrhea, rash, and hyperglycemia may require proactive management. This article provides practical recommendations to support prevention and early intervention to optimize adherence and treatment outcomes.

## Introduction

The PI3K/AKT pathway regulates cellular growth, proliferation, and survival. It is overactivated by genetic alterations in several cancers, including ~50% of hormone receptor (HR)-positive/human epidermal growth receptor 2 (HER2)-negative breast cancer (BC) cases^1^. Activating mutations in the phosphatidylinositol-4,5-bisphosphate 3-kinase catalytic alpha subunit (*PIK3CA*) gene occur in 30% to 40% of cases^2,3^, an activating mutation in the AKT serine/threonine kinase 1 (*AKT1*) gene occurs in around 5% of cases^4^, and loss-of-function inactivating mutations or deletions of phosphatase and tensin homolog *(PTEN)* occur in 5% to 10% of cases^1,2,5,6^. Hyperactivation of the PI3K/AKT pathway, caused by these tumor alterations, is associated with endocrine therapy (ET) resistance and disease progression. Inhibition of AKT, the master switch of the pathway, restricts oncogenic signaling from PI3K, AKT, and PTEN, before those signals can have further downstream effects that drive tumor growth^7–9^. Several agents, namely, alpelisib, capivasertib, everolimus and inavolisib, have been developed to inhibit key proteins within this pathway to overcome ET resistance and arrest cancer progression^10^.

Capivasertib targets all 3 AKT isoforms (AKT 1/2/3), inhibiting signaling driven by *PIK3CA, AKT1,* and/or *PTEN* alterations. In combination with fulvestrant, capivasertib is approved in the US for the treatment of adult patients with HR-positive/HER2-negative, locally advanced, or metastatic BC with ≥1 *PIK3CA*, *AKT1*, and/or *PTEN* alterations, following disease progression on ≥1 ET-based regimen in the metastatic setting or recurrence on or within 12 months of completing adjuvant ET^11–14^. The phase III CAPItello-291 trial demonstrated significant improvement in progression-free survival in a *PIK3CA/AKT1/PTEN*-altered population with capivasertib + fulvestrant (*n* = 155) vs placebo + fulvestrant (*n* = 134; 7.3 months vs 3.1 months; hazard ratio, 0.50; *P* < 0.001)^11^. However, as capivasertib inhibits physiologic PI3K/AKT pathway signaling, it leads to on-target, off-tumor toxicities^15^. The most common adverse events (AEs) reported in the safety population for capivasertib (*n* = 355) and placebo (*n* = 350) were diarrhea, cutaneous adverse reactions (CARs; group term of butterfly rash, dermatitis, allergic dermatitis, dry skin, eczema, erythema multiforme, hand dermatitis, palmar-plantar erythrodysesthesia syndrome, pruritus, rash, erythematous rash, maculopapular rash, papular rash, skin discoloration, skin fissures, skin reaction, skin ulcer, urticaria, purpura, erythema, and drug eruption^11^), and elevated random glucose levels^11^; the most frequently reported AEs of grade ≥3 were rash (group term of rash, rash macular, maculopapular rash, rash papular, and rash pruritic^11^; 12.1%), diarrhea (9.3%), and hyperglycemia (2.0%)^16,17^. Serious AEs were reported by 57 (16.1%) patients receiving capivasertib and 28 (8.0%) patients receiving placebo. Dose interruptions, reductions, and permanent treatment discontinuations due to AEs occurred in 38.9%, 19.7%, and 13.0% of patients receiving capivasertib, and 12.3%, 1.7%, and 2.3% of patients receiving placebo, respectively^18^.

This article reviews the monitoring and management considerations for common capivasertib-associated AEs, specifically, diarrhea, rash, and hyperglycemia. While the prescribing information (PI) offers guidance on dosage modifications, we provide structured recommendations for toxicity management, informed by clinical trial data and real-world experience, to support patient care.

## Monitoring and management of capivasertib-associated adverse events

### Diarrhea

#### Etiology and incidence

Diarrhea is a common toxicity resulting from PI3K/AKT pathway inhibition, which may be due to decreased AKT/mammalian target of rapamycin-dependent activation of Na^+^/H^+^ exchanger 3, impairing intestinal sodium absorption and water homeostasis^19–21^. In CAPItello-291, 72.4% (257/355) of patients receiving capivasertib experienced diarrhea, with a median time to onset of 8 days (range, 2–22 days), with a majority of cases categorized as intermittent or short-term (<4 days)^17^. Grade 3 diarrhea occurred in 9.3% (33/355) of patients. Treatment discontinuation due to diarrhea was reported in 2% of patients, dose reduction in 8% of patients, and dose interruptions in 10% of patients^11,17^. Primary prophylaxis for diarrhea was not a protocol requirement in CAPItello-291; secondary prophylaxis with antidiarrheal treatment (i.e., loperamide) was recommended at the discretion of the healthcare provider (HCP). The median time to improvement by ≥1 grade for grade ≥2 diarrhea was 4 days (range, 1–154 days)^11^.

#### Management of capivasertib-associated diarrhea

**Fig. 1:**
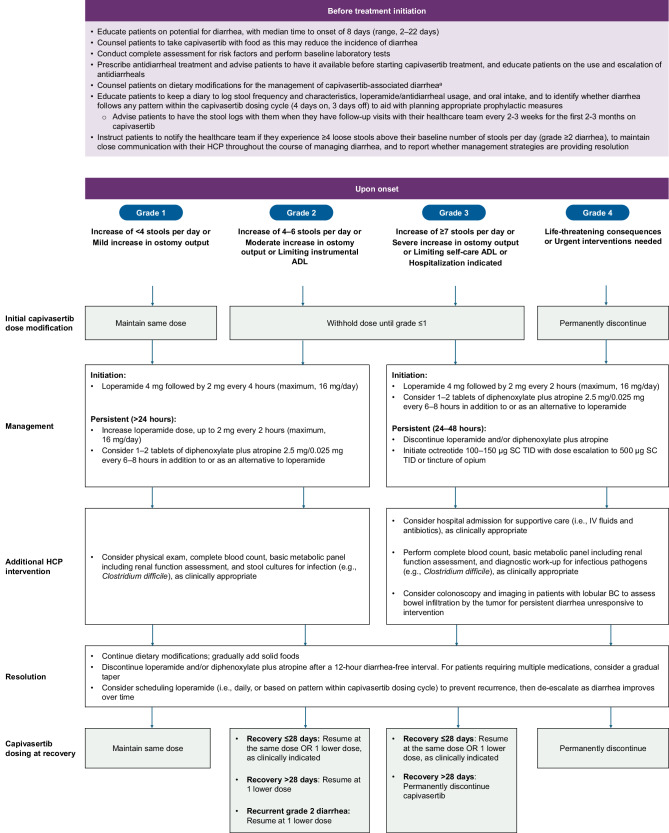
Algorithm for the monitoring and management of capivasertib-associated diarrhea^11,16,27,28,31,72^. Shaded boxes represent recommended dosage modifications from the capivasertib PI. ^a^Refer to Table 2 for dietary strategies to manage capivasertib-induced diarrhea. Counsel patients on the dietary modifications, such as eating frequent, small meals (e.g., bananas, applesauce, toast); ensuring adequate hydration with clear liquids (e.g., water, broth); and avoiding or limiting high-fiber foods (e.g., raw vegetables, beans, whole grains), lactose-containing products, caffeinated and alcoholic beverages, and high-osmolar supplements. ADL activities of daily living, BC breast cancer, GI gastrointestinal, HCP healthcare provider, IV intravenous, PI prescribing information, SC subcutaneously, TID 3 times per day.

Integrated recommendations, including capivasertib dose modifications and interruptions from the PI, and additional management strategies for diarrhea are summarized in Table 1 and Fig. 1.

**Table 1:** Summary of recommendations for the management of capivasertib-associated adverse events

Summary recommendations to address diarrhea
1. Educate patients to recognize low-grade diarrhea versus symptoms that require enhanced supportive care.
2. Screen for risk factors and use a stool diary.
3. Manage diarrhea with a step-wise approach including dietary counseling, antidiarrheal agents escalated as necessary, and capivasertib dose modifications.
**Summary recommendations to address rash**
4. Educate patients to recognize capivasertib-associated rash and other symptoms that require enhanced supportive care.
5. Implement rash prophylaxis with skin protective measures and a nonsedating H1 antihistamine.
6. Estimate BSA involved by rash to select interventions, including antihistamines, topical or systemic steroids, and capivasertib dose modifications.
**Summary recommendations to address hyperglycemia**
7. Identify candidates for capivasertib treatment during first-line metastatic breast cancer treatment or earlier to optimize glycemic status.
8. Screen for hyperglycemia risk factors and provide glucose monitoring education that aligns with the clinic team’s workflows for alerts, triage, and management.
9. Implement dietary modifications and physical activity prior to and during capivasertib treatment.
10. Consider hyperglycemia prophylaxis with metformin for patients at elevated risk (i.e., HbA_1c_ ≥ 5.7%, prediabetes, BMI ≥ 30 kg/m^2^, elevated baseline FG); start metformin or escalate existing dose and add other agents to treat hyperglycemia, as needed, while avoiding insulin secretagogues, which can cause hypoglycemia.

*BMI* body mass index, *BSA* body surface area, *FG* fasting glucose, *HbA*_1c_ hemoglobin A1C.

*Recommendation 1: Educate patients to recognize low-grade diarrhea versus symptoms that require enhanced supportive care*. Before starting capivasertib, inform patients about capivasertib-associated diarrhea, which is typically mild to moderate and likely to occur within the first 1–2 weeks, and about symptoms that may require immediate medical attention, such as fever (≥100.5 °F/38 °C), severe abdominal cramping, or signs of dehydration^17^. Counsel patients to take capivasertib with food, as this may reduce the incidence of diarrhea^22^ and to notify the healthcare team if experiencing ≥4 loose stools per day above their baseline number (i.e., grade ≥2 diarrhea).

*Recommendation 2: Screen for risk factors and use a stool diary*. Screen patients for a history of diarrhea, concomitant medications (e.g., laxatives, magnesium supplements, glucagon-like peptide-1 receptor [GLP-1R] agonists, metformin), and comorbidities that may increase risk or worsen diarrhea^23^. Assess renal function and electrolytes at baseline and identify patients who may be at higher risk of developing dehydration^24,25^.

Encourage patients to use a diary to log stool frequency and characteristics, loperamide/antidiarrheal usage, and oral intake to help with symptom triage and identifying whether diarrhea follows a pattern within the capivasertib dosing cycle (4 days on, 3 days off) that could be addressed with prophylactic measures^26^. Advise patients to bring stool logs to follow-up visits with their healthcare team every 2–3 weeks for the first 2–3 months on capivasertib and to contact their HCP between visits, as needed.

*Recommendation 3: Manage diarrhea with a step-wise approach including dietary counseling, capivasertib dose modifications, and antidiarrheal agents escalated as necessary*. When patients report capivasertib-associated diarrhea, counsel them on dietary modifications, such as eating frequent, small meals (i.e., bananas, applesauce, and toast); ensuring adequate hydration with clear liquids such as water or broth; and avoiding or limiting high-fiber foods such as raw vegetables, beans, and whole grains, lactose-containing products, caffeinated and alcoholic beverages, and high-osmolar supplements, as outlined in Table 2^27–29^.

**Table 2:** Dietary strategies for the management of capivasertib-induced diarrhea^27–29^

Choose	Limit/Avoid
• Frequent, small meals • Buttermilk, skim milk, low-fat milk, plant-based milk, low-fat cheese • Well-cooked vegetables or fruits without skin or seeds • Bananas, applesauce, juice without pulp • Bread, pasta, noodles made with refined white flour, white rice • Cereals with <2 g of fiber per serving, as indicated on the product’s nutritional label • Tender well-cooked meat, fish, eggs • Drink 8–10 large glasses of clear liquids/day (e.g., water, oral rehydration solutions, broth, decaffeinated coffee or tea)	• Lactose-containing products (e.g., whole milk, half-and-half, sour cream) • Salads • Raw vegetables and raw fruits (e.g., apples, pears, cherries, mangoes, oranges) • Dry fruits (e.g., prunes, raisins) • High-fiber vegetables (e.g., beets, brussels sprouts, broccoli, corn, beans, cabbage, cauliflower, peas) • Whole wheat and whole grains, brown rice, any bread/cereal with fruits or nuts • Lentils, lima beans, chickpeas, kidney beans, black beans • Whole nuts • Caffeine-containing beverages • Alcoholic beverages • High-osmolar supplements (e.g., liquid nutritional supplements such as Ensure^®^, Boost^®^; electrolyte drinks such as Pedialyte^®^; protein powders; or fiber supplements)

For grade 2 diarrhea (increase of 4–6 stools/day over baseline, or moderate increase in ostomy output, or limiting instrumental activities of daily living [ADL]) or grade 3 diarrhea (increase of ≥7 stools/day over baseline; hospitalization indicated; severe increase in ostomy output compared to baseline; or limiting self-care ADL), advise patient to withhold capivasertib until recovery to grade ≤1 diarrhea. If recovery occurs in ≤28 days, resume capivasertib at the same dose or one lower dose as clinically indicated. For grade 2 diarrhea, if recovery occurs in >28 days, then resume as outlined in Fig. 1. For grade 3 diarrhea persisting >28 days or for grade 4 diarrhea (life-threatening consequences or urgent intervention indicated), permanently discontinue capivasertib^11,30^. Instruct patients to initiate treatment with loperamide (4 mg followed by 2 mg every 4 h; maximum, 16 mg/day) at first sign of diarrhea as outlined in Fig. 1. Most diarrheal episodes are expected to be manageable with loperamide, escalating the dose, as needed. Additional agents, like diphenoxylate/atropine or octreotide, can be considered if diarrhea persists for >24 h despite maximal use of loperamide^27,31^. If diarrhea resolves, which generally occurs during the 3 days per week off capivasertib, instruct patients to continue dietary modifications and discontinue or gradually taper antidiarrheals after a 12-h diarrhea-free interval^27,30^. If resuming capivasertib at the same dose, consider scheduling loperamide to prevent recurrence, then de-escalate as diarrhea improves.

If patients develop severe or persistent diarrhea despite dose interruption and antidiarrheal treatment, consider hospitalization for supportive care with intravenous (IV) hydration, electrolyte repletion, and antibiotics, as well as multidisciplinary care. Perform a diagnostic work-up, including diagnostic testing for infectious pathogens (e.g., *Clostridium difficile, Salmonella* spp., *Campylobacter* spp., *Giardia* spp., *Entamoeba* spp., *Cryptosporidium* spp., *Shigella* spp., *Escherichia coli*), complete blood count, and basic metabolic panel, including assessment of renal function^27,32,33^. Consider subcutaneous (SC) octreotide 100–150 µg 3 times per day (TID), with dose escalation as needed up to 500 µg SC TID. Additional antidiarrheal medications, such as tincture of opium, can also be considered^27^. In patients with lobular BC who may harbor gastrointestinal (GI) metastases, colonoscopy or other imaging methods may be needed to assess for bowel infiltration, which may mimic or exacerbate treatment-related diarrhea and require tailored oncologic management^34^.

### Rash

#### Etiology and incidence

The PI3K/AKT pathway plays a key role in skin homeostasis, supporting the epidermal barrier, hair follicle regeneration, wound healing, and skin senescence^35^. Although the exact etiology of rash associated with PI3K/AKT inhibitors is not fully understood, it is considered an “on-target off-tumor” effect of pathway inhibition. Evidence suggests involvement of histamine-producing cells and eosinophils, and that both direct pathway inhibition and immune-mediated mechanisms may be involved^36,37^.

In CAPItello-291, rash (preferred terms of rash, macular rash, maculopapular rash, papular rash, and pruritic rash) occurred in 38.0% (135/355) of patients receiving capivasertib + fulvestrant, with a median time to onset of 12 days (range, 10–15 days). Approximately 75% of rash events were reported within the first treatment cycle. Grade 3 rash (macules/papules covering >30% body surface area [BSA] with moderate or severe symptoms or limiting self-care ADL) occurred in 12.1% (43/355) of patients^17^. Drug reaction with eosinophilia and systemic symptoms occurred in 0.3% of patients. Treatment discontinuation due to rash was reported in 4.5% of patients, dose reduction in 4.5% of patients, and dose interruptions in 11.8% of patients^11,17^. Primary prophylaxis with an antihistamine to reduce the incidence of rash was not a protocol requirement in CAPItello-291^17^. The median time to ≥1-grade improvement for grade ≥2 CARs was 12 days (range, 2–544 days)^11^.

#### Prevention and management of capivasertib-associated rash

**Fig. 2:**
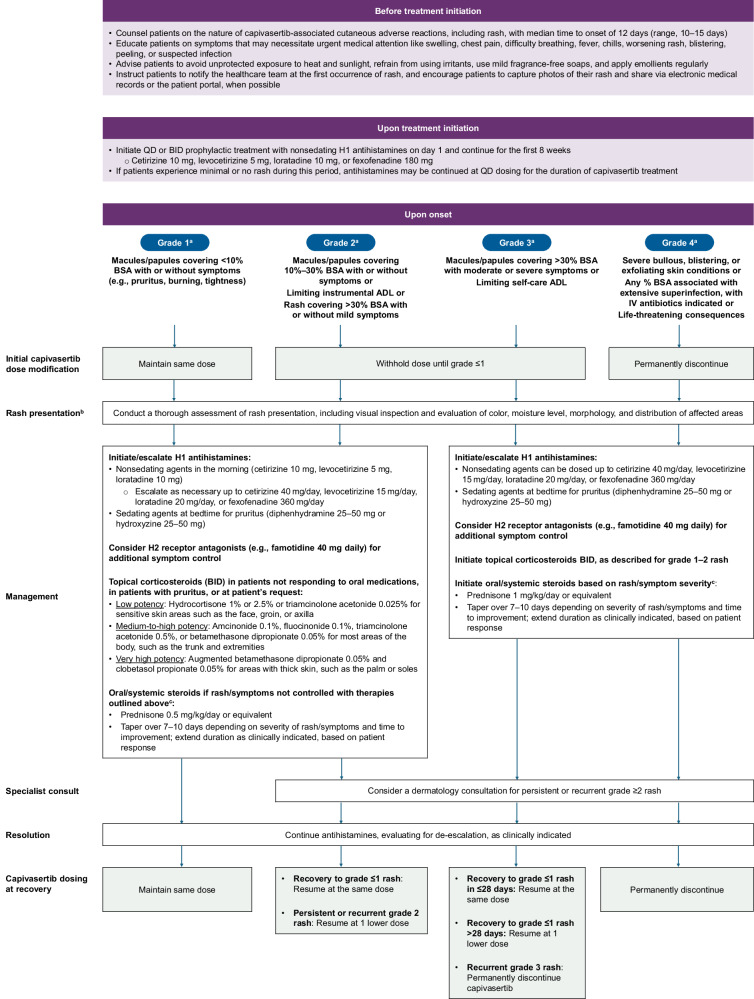
Algorithm for the monitoring and management of capivasertib-associated rash^11,15,16,30,38,39,43,46^. Shaded boxes represent recommended dosage modifications from the capivasertib PI. The PI recommendations provided encompass the group term of CARs, which includes rash in addition to butterfly rash, dermatitis, allergic dermatitis, dry skin, eczema, erythema multiforme, hand dermatitis, palmar-plantar erythrodysesthesia syndrome, pruritus, erythematous rash, maculopapular rash, papular rash, skin discoloration, skin fissures, skin reaction, skin ulcer, urticaria, purpura, erythema and drug eruption. ^a^Grading is for maculopapular rash. Refer to CTCAE v5.0 for grading other dermatologic adverse events. ^b^Estimate BSA involved using tools like the palm method (one palm, including fingers, equals 1% BSA). ^c^Use caution with systemic corticosteroids due to the potential for hyperglycemia, which may necessitate increasing frequency of glucose monitoring 1–2 days after initiation of steroids, and close follow-up thereafter. ADL activities of daily living, BID twice daily, BSA body surface area, CARs cutaneous adverse reactions, CTCAE Common Terminology Criteria for Adverse Events, IV intravenous, PI prescribing information, QD once daily.

Guidance from the PI pertaining to capivasertib dose modifications, interruptions, and additional management strategies for rash based on our experience and expertise is summarized in Table 1 and Fig. 2.

*Recommendation 4: Educate patients to recognize capivasertib-associated rash and other symptoms that require enhanced supportive care*. Inform patients that capivasertib-associated rash is likely to occur within the first treatment cycle and instruct them to notify the healthcare team at the first occurrence. Emphasize that their HCP should be immediately notified about any swelling, chest pain, shortness of breath, fever, chills, worsening rash, blistering, peeling, or suspected infection^38^. Encourage patients to share photos of their rash via electronic medical records or patient portal for real-time assessment, when possible.

*Recommendation 5: Implement rash prophylaxis with skin protective measures and a nonsedating H1 antihistamine*. Advise patients to avoid unprotected exposure to heat and sunlight, refrain from using possible skin irritants, use mild fragrance-free soaps, and apply emollients regularly^23^. We recommend initiating a prophylactic nonsedating H1 antihistamine (e.g., cetirizine 10 mg, levocetirizine 5 mg, loratadine 10 mg, or fexofenadine 180 mg) once daily (QD) or twice daily (BID) starting on day 1 of capivasertib treatment and continuing for the first 8 weeks of therapy. If patients experience minimal or no rash during this period, antihistamines may be continued QD for the duration of capivasertib treatment^17,18,23,39^.

*Recommendation 6: Estimate BSA involved by rash to select interventions, including antihistamines, topical or systemic steroids, and capivasertib dose modifications.* In patients presenting with capivasertib-associated rash, conduct a visual inspection of rash characteristics (color, moisture, morphology, and distribution) and consider treatment strategies by grade as outlined in Fig. 2. Estimate BSA involved using tools like the palm method (one palm, including fingers, equals 1% BSA)^23,40,41^.

Grade 1 rash (macules/papules covering <10% BSA with or without symptoms [e.g., pruritus, burning, tightness]) can typically be managed with H1 antihistamines, H2 receptor antagonists, and/or topical corticosteroids^15,30,39,42^. Consider utilizing sedating H1 antihistamines (e.g., diphenhydramine, hydroxyzine) at bedtime or adding an H2 receptor antagonist (e.g., famotidine 40 mg QD) for symptomatic control of rash, including pruritus^43^. Although H2 receptor antagonists are not primarily used to treat rash, they may provide symptomatic relief of pruritus when used adjunctively with H1 antihistamines. In a randomized, controlled study, famotidine significantly reduced pruritus in patients with urticaria, similar to diphenhydramine but without sedating side effects^44^. Topical corticosteroids BID can also be used to treat grade 1 rash, based on the extent of the rash and pruritus. The choice of topical corticosteroids should be guided by the location of the rash. Low-potency steroids such as hydrocortisone 1% or 2.5% or triamcinolone acetonide 0.025% are generally appropriate for use on areas with thinner, more sensitive skin, such as the face, groin, or axilla. In contrast, medium- to high-potency steroids such as amcinonide 0.1%, fluocinonide 0.1%, triamcinolone acetonide 0.5%, or betamethasone dipropionate 0.05%, are appropriate for use on most areas of the body, such as the trunk and extremities. Very high-potency agents such as augmented betamethasone dipropionate 0.05% and clobetasol propionate 0.05% are used for areas with thick skin, such as the palms or soles, and should be avoided on sensitive areas^39,45^. Depending on the severity of the rash and/or associated symptoms, a brief course of a systemic corticosteroid (tapered over 7 to 10 days), such as prednisone or methylprednisone, could be utilized for symptomatic grade 1 rash not controlled with the therapies outlined above. If utilizing oral steroids, fasting glucose (FG) monitoring should be intensified, with additional glucose testing 1–2 days after initiation of steroids and close follow-up thereafter. Additional consideration should be given for temporary interruption of capivasertib. We have found that patients who experience low-grade rash or other symptoms during the 4 days on capivasertib treatment may have significant recovery during the 3 days off. As such, consider a short delay in starting the next capivasertib dosing week as a strategy to manage grade 1 rash.

For patients with grade 2 rash (macules/papules covering 10–30% BSA with or without symptoms or rash-limiting instrumental ADL or a rash covering >30% BSA with or without mild symptoms), withhold capivasertib until recovery to grade ≤1, then resume as outlined in Fig. 2^11,30^. Initiate or escalate treatment with H1 antihistamines, in addition to adding an H2 receptor antagonist and/or topical corticosteroids BID to manage rash/pruritis^39,43^. Consider a course of oral/systemic steroids, such as prednisone 0.5 mg/kg/day or equivalent, tapered over 7–10 days, depending on the severity of the rash or symptoms and time to improvement; steroid course can be extended beyond 10 days as needed^46^. Use caution with systemic corticosteroids due to potential for hyperglycemia and associated acute complications; increase glucose monitoring frequency 1–2 days after steroid initiation, with close follow-up^47,48^.

For patients with grade 3 rash, withhold capivasertib until recovery to grade ≤1, then resume as outlined in Fig. 2^11^. Consider topical steroids BID, intensified antihistamine dosing, and oral/systemic corticosteroids (prednisone 1 mg/kg/day or equivalent)^39,46^. The dose of the oral/systemic corticosteroid should be tapered over 7–10 days depending on rash severity or symptoms and time to improvement^39^. Permanently discontinue capivasertib for recurrent grade 3 rash. For grade 4 rash (severe bullous, blistering, or exfoliating skin conditions; any % BSA associated with superinfection requiring IV antibiotics), permanently discontinue capivasertib and assess the need for hospitalization^11^.

Consider dermatology consultation for persistent or recurrent grade ≥2 rash^39^. Emerging therapies targeting eosinophilic inflammation are under development for rash associated with PI3K inhibitors and may offer additional management options in the future^49^.

### Hyperglycemia

#### Etiology and incidence

**Fig. 3:**
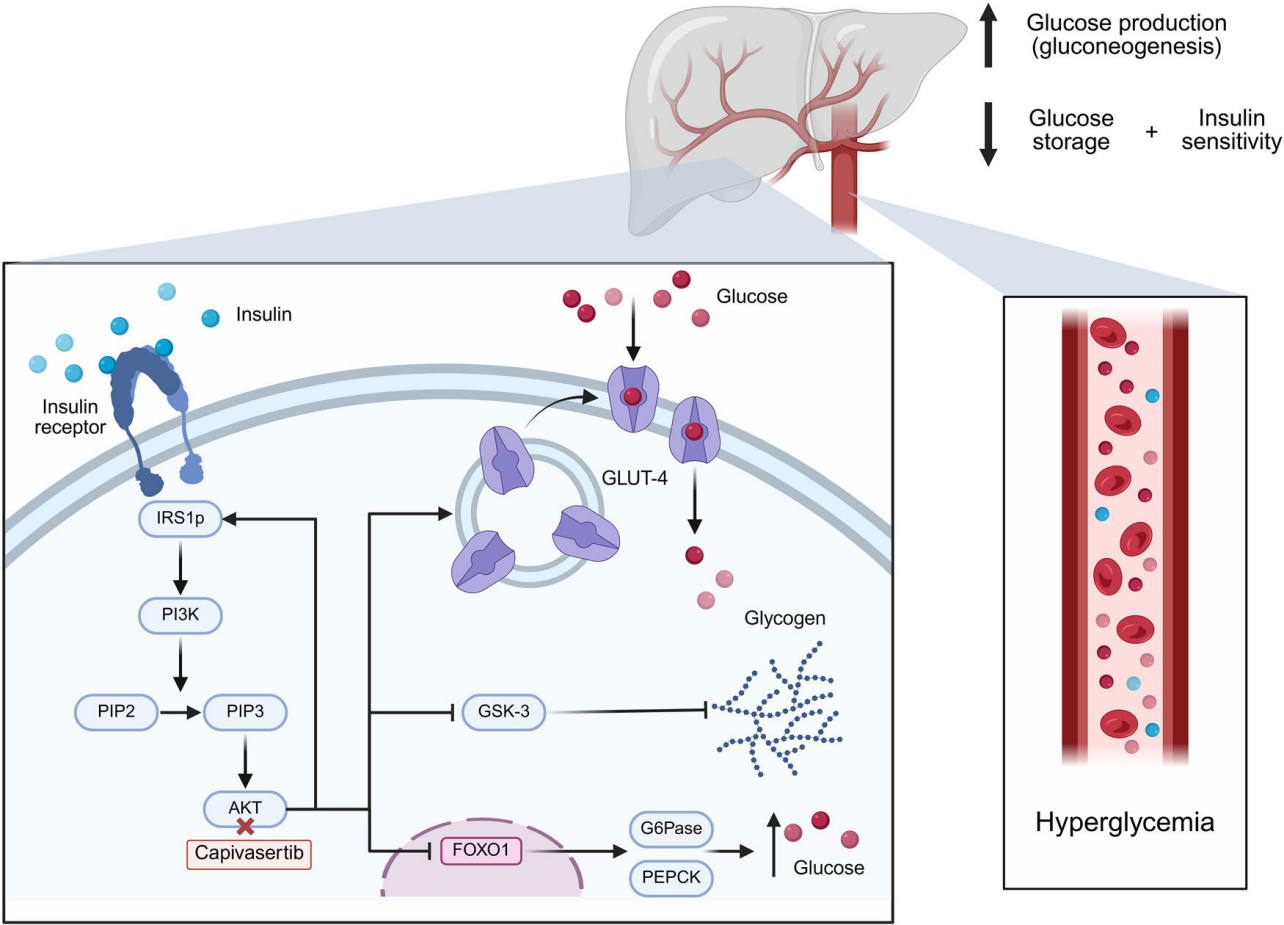
Mechanism of AKT inhibitor–induced hyperglycemia^73^. This figure was created in https://BioRender.com. The left panel shows normal insulin signaling and glucose regulation. Under normal conditions, insulin binding to its receptor initiates a signaling cascade through IRS1p and PI3K, which facilitates the conversion of PIP2 to PIP3, activating AKT. AKT promotes GLUT-4 translocation to the cell membrane, allowing for cellular glucose uptake. AKT also facilitates glycogen synthesis via inhibition of GSK3 and suppresses hepatic gluconeogenesis by inhibiting FOXO1, which downregulates G6Pase and PEPCK. When AKT is inhibited, GLUT–4–mediated glucose uptake is impaired, and FOXO1 is activated. This leads to increased hepatic glycogenolysis and gluconeogenesis, resulting in systemic hyperglycemia, as indicated in the right panel. AKT protein kinase B, FOXO1 forkhead box protein O1, G6Pase glucose-6-phosphatase, GSK-3 glycogen synthase kinase 3, GLUT-4 glucose transporter type 4, IRS1p phosphorylated insulin receptor substrate 1, PEPCK phosphoenolpyruvate carboxykinase, PI3K phosphoinositide 3-kinase, PIP2 phosphatidylinositol-4,5-bisphosphate, PIP3 phosphatidylinositol-3,4,5-trisphosphate.

The PI3K/AKT signaling pathway regulates glucose homeostasis by mediating insulin signaling via the insulin receptor through this pathway to promote glucose uptake, suppress gluconeogenesis, and enhance glycogenesis^50^. AKT facilitates the fusion of glucose transporter type 4 (GLUT4)-containing vesicles with the cell membrane, increasing glucose uptake^51^. PI3K/AKT pathway inhibition disrupts these processes, leading to reduced glucose uptake, increased gluconeogenesis, and impaired insulin signaling, making hyperglycemia a common side effect of PI3K/AKT inhibitors (Fig. 3)^15,51–54^.

Patients with type 2 diabetes (hemoglobin A1C [HbA_1c_], 6.5–8.0%) were included in CAPItello-291, while patients with uncontrolled (HbA_1c_, ≥8.0%) and/or insulin-dependent diabetes at baseline were ineligible. Elevation of FG from baseline occurred in 37% of capivasertib-treated patients, including 11% with grade 2 (FG, >160–250 mg/dL), 2% with grade 3 (FG, >250–500 mg/dL), and 1.1% with grade 4 (FG, >500 mg/dL) events^11^. Median time to onset of hyperglycemia was 15 days (range, 1–51 days)^17^. Diabetic ketoacidosis (DKA) occurred in 0.3% of patients and diabetic metabolic decompensation in 0.6% of patients^11^. Treatment discontinuation due to hyperglycemia occurred in 0.3% of patients, dose reduction in 0.6% of patients, and dose interruptions in 2.5% of patients^17^.

Post-marketing cases of severe hyperglycemia, including DKA and hyperglycemic hyperosmolar syndrome, have been reported with capivasertib^55,56^. These reports occurred in the context of severe infection and/or multiorgan dysfunction, highlighting the importance of monitoring for hyperglycemia in the context of infection and other serious illnesses. A fatal case of DKA occurred in a patient who presented with urinary tract infection, septic shock, and acute renal failure, with severe infection and associated complications likely contributing to DKA and the fatal outcome^55^.

#### Prevention and management recommendations

**Fig. 4:**
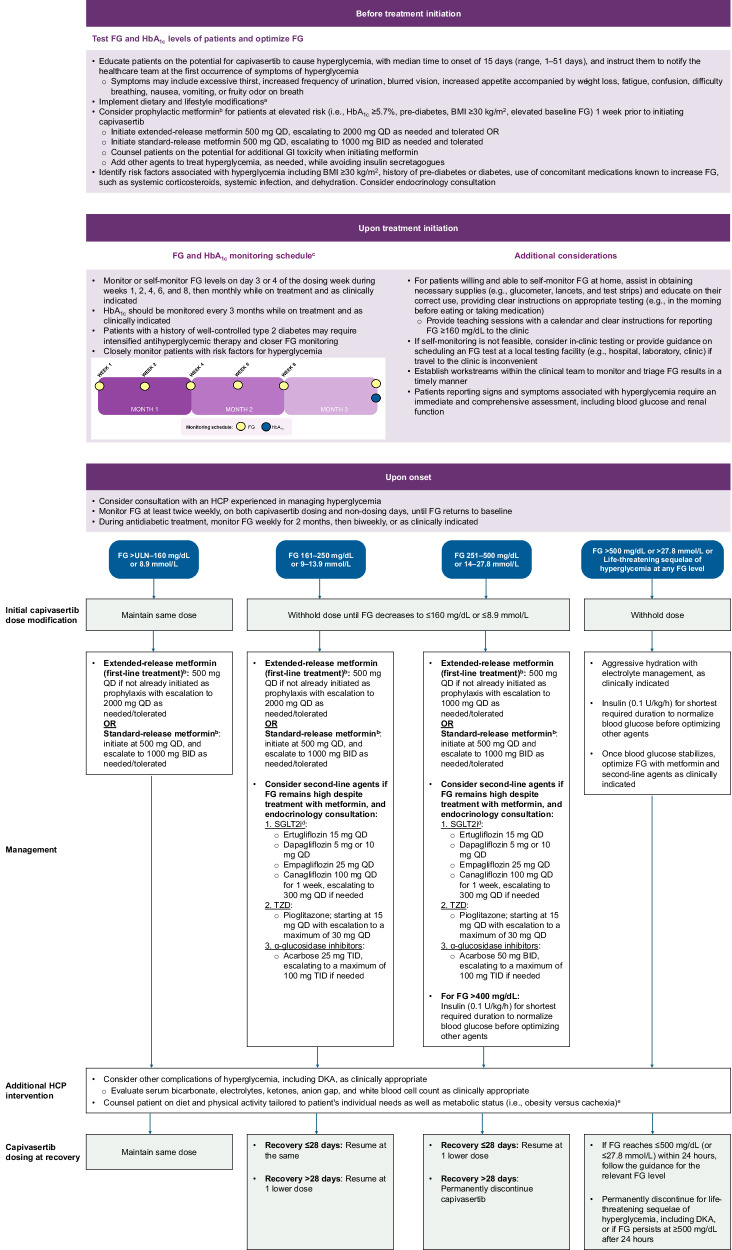
Algorithm for the monitoring and management of capivasertib-associated hyperglycemia^11,15,30,39,55^. Shaded boxes represent recommended dosage modifications from the capivasertib PI. ^a^Engage in at least 30 min of low-to-moderate physical activity daily. Maintain a healthy diet (e.g., low-carbohydrate [<100 g/day], low-fat, and high-fiber diet; small, frequent meals and snacks; balancing carbohydrates over the course of the day). Avoid substantial weight gain and limit alcohol consumption. If needed, recommend a ketogenic diet (total carbohydrate intake of <50 g/day). Diet and physical activity recommendations should be tailored to the needs of the individual patient and should consider metabolic status (i.e., obesity versus cachexia). ^b^In patients with eGFR <30 mL/min/1.73 m^2^, or as indicated per local institutional protocols, withhold metformin prior to and 48 h after contrast imaging to minimize the risk of acute kidney injury. ^c^Refer to Table 3 for the schedule of monitoring of FG and HbA_1c_ levels in patients treated with capivasertib. ^d^Monitor for euglycemic DKA (checking urine or blood ketone levels) in patients receiving SGLT2i and advise urgent care if DKA symptoms appear. ^e^Refer to Table 4 for dietary strategies to manage capivasertib-induced hyperglycemia. BC breast cancer, BG blood glucose, BID twice daily, BMI body mass index, DKA diabetic ketoacidosis, eGFR estimated glomerular filtration rate, FG fasting glucose, HbA_1c_ hemoglobin A1C, HCP healthcare provider, HG hyperglycemia, QD once daily, PI prescribing information, SGLT2i sodium-glucose cotransporter 2 inhibitor, TZD thiazolidinedione, ULN upper limit of normal.

Integrated recommendations, including guidance from the PI on capivasertib dose modifications, interruptions, and monitoring and management strategies for hyperglycemia, based on our experience and expertise, are summarized in Table 1 and Fig. 4.

*Recommendation 7: Identify candidates for capivasertib treatment during first-line metastatic BC treatment or earlier to optimize glycemic status*. Identifying patients who may benefit from capivasertib after progression on first-line ET (i.e., those whose cancer has ≥1 *PIK3CA*, *AKT1*, and/or *PTEN* alterations), will provide an opportunity to achieve glycemic control, if needed, before initiating capivasertib. Management strategies to achieve glycemic control include lifestyle modifications and endocrinology consultation for patients with prediabetes/diabetes. Monitoring and controlling FG levels during first-line therapy can help ensure that patients transition to capivasertib with stable glycemic control, which may enable treatment persistence. Prior to initiating capivasertib, it is important to evaluate and treat preexisting hyperglycemia and ensure that HbA_1c_ < 8%.

*Recommendation 8: Screen for hyperglycemia risk factors and provide glucose monitoring education that aligns with the clinic team’s workflows for alerts, triage, and management*. Risk factors for hyperglycemia include body mass index (BMI) ≥ 30 kg/m^2^, history of prediabetes/diabetes, and use of concomitant medications known to increase FG, such as systemic corticosteroids, systemic infection, and dehydration^16,39,57^. Consider endocrinology consultation for these patients. Educate patients on the potential for capivasertib to cause hyperglycemia, including signs and symptoms.

After starting capivasertib treatment, monitor FG pre-dose on day 3 or 4 of the treatment week during weeks 1, 2, 4, 6, and 8, and then monthly thereafter (Table 3). The recommendation to monitor FG on day 3 or 4 of the treatment week is based on pharmacokinetic data, accounting for the impact of capivasertib plasma levels on FG^11^. For patients able to self-monitor at home, assist in obtaining necessary supplies (e.g., glucometer, lancets, test strips) and educate on their correct use, providing clear instructions on appropriate testing (e.g., in the morning before eating or taking medication). Provide teaching sessions with a calendar to track FG levels along with instructions for when and how to report results to the clinic. Patients should be instructed to notify the healthcare team for an FG level ≥160 mg/dL. If self-monitoring is not feasible, consider in-clinic testing or provide guidance on scheduling an FG test at a local testing facility (e.g., hospital, laboratory, clinic) if travel to the clinic is inconvenient. Establish workstreams within the clinical team to monitor, triage, and manage elevated FG results. Educate patients about the signs and symptoms associated with hyperglycemia, including excessive thirst, increased urination frequency, blurred vision, increased appetite, weight loss and fatigue, as well as confusion, difficulty breathing, nausea, vomiting, or fruity odor on breath, the latter which require immediate medical evaluation.

**Table 3:** Schedule of monitoring of FG and HbA_1c_ levels in patients treated with capivasertib^11,58^

	Recommended schedule for the monitoring of FG and HbA_1c_ levels
Before initiating treatment with capivasertib	Test for FG levels and HbA_1c_ levels, and optimize FG
Upon initiating treatment with capivasertib	Monitor or self-monitor FG levels on day 3 or 4 of the dosing week during weeks 1, 2, 4, 6, and 8, then monthly while on treatment, and as clinically indicated HbA_1c_ should be monitored every 3 months while on treatment and as clinically indicated Closely monitor FG levels in patients with a history of well-controlled type 2 diabetes
If hyperglycemia develops after initiating treatment with capivasertib	Monitor FG at least twice weekly, on days on and off capivasertib treatment, until FG decreases to baseline levels^a^ During treatment with antidiabetic medication, FG should be monitored at least once a week for 2 months, followed by once every 2 weeks or as clinically indicated^a^

*FG* fasting glucose, *HbA*_*1c*_ hemoglobin A_1c_, *HCP* healthcare provider.

^a^It is recommended to test FG pre-dose on day 3 or 4 of the dosing week.

*Recommendation 9: Implement dietary modifications and physical activity prior to and during capivasertib treatment*. Dietary modifications play an important role in preventing and managing capivasertib-associated hyperglycemia. Patients should be encouraged to follow a low-carbohydrate, high-fiber diet, engage in regular physical activity, avoid substantial weight gain, and limit alcohol consumption. A summary of dietary recommendations is provided in Table 4^58,59^. Diet and physical activity recommendations should be tailored to individual patient needs and should consider metabolic status (i.e., obesity versus cachexia).

**Table 4:** Dietary strategies for the management of capivasertib-induced hyperglycemia^59,71^

Food type/strategy	Choose	Avoid
Beverages	Sugar-free drinks, plain tea or coffee, seltzer, sugar-free instant breakfast (with sugar substitutes)	Fruit juice (more than ½ cup/day), sugar-sweetened beverages, flavored drinks, milkshakes, sports drinks, alcoholic beverages
Breads and cereals	Whole-grain breads and cereals	Sweetened cereals, granola bars, breakfast bars, pastries, doughnuts; limit rice and pasta
Fruits	Fresh or frozen fruits without added sugar, fruits canned in water or juice, fruits with sugar substitutes	Fruits canned in syrup, sugar-sweetened fruits; limit 100% fruit juice to ½ cup/day
Vegetables	Fresh or frozen non-starchy vegetables	Starchy vegetables such as potatoes, corn, beans, and peas (limit portions)
Meat and meat substitutes	Beef, pork, chicken, fish, eggs, beans, shellfish, cheese, tofu	High-fat/sodium meats like cold cuts, bacon, sausage, fried meats, hot dogs
Milk	Low-fat milk, buttermilk, plain or light yogurt, soymilk	Flavored or sweetened milk and yogurt
Desserts and snacks	Sugar-free jelly, pudding, syrup, ice cream, angel food cake, plain graham crackers, sugar substitutes (e.g., Splenda, Equal)	Regular sugar, honey, syrup, candies, baked goods, ice cream, jam, pudding, marshmallows, condensed milk, sorbet
Ketogenic diet	Focus on a high-fat (70–80%), moderate-protein (10–20%), and very-low-carbohydrate (5–10%) intake Examples: Avocados; dark chocolate (90% or higher cocoa solids); nuts such as macadamia, walnuts, almonds, pecans; seeds such as sunflower, pumpkin, sesame, hemp, flax; plant fats like olive, palm, coconut oil; fatty fish; eggs; non-starchy vegetables	Grains such as bread, rice, pasta, sugar, and sweetened foods or drinks, high-carbohydrate fruits, starchy vegetables, and sweetened dairy products

*Recommendation 10: Consider hyperglycemia prophylaxis with metformin for patients at elevated risk (i.e., HbA*_*1c*_ ≥ *5.7*%*, prediabetes, BMI* ≥ *30* kg/m^2^*, elevated baseline FG); start metformin or escalate existing dose and add other agents to treat hyperglycemia, as needed, while avoiding insulin secretagogues, which can cause hypoglycemia*. Prophylactic use of insulin sensitizers, such as metformin, can mitigate PI3K/AKT inhibitor–induced hyperglycemia. In the METALLICA trial, for patients with HR-positive/HER2-negative advanced BC and normal or prediabetic status (baseline HbA_1c_, <6.5%) receiving alpelisib + ET, prophylactic metformin (500 mg BID, escalating to 1000 mg BID if no GI intolerance occurred) significantly reduced hyperglycemia risk^60^. Therefore, we recommend initiating prophylactic extended-release metformin 500 mg/day one week before initiating capivasertib, escalating to 2000 mg/day, as needed and as tolerated for patients with an elevated risk of developing hyperglycemia (i.e., HbA_1c_ ≥ 5.7%, prediabetes, BMI ≥ 30 kg/m^2^, elevated baseline FG). Standard-release metformin can be utilized at a dose of 500 mg/day, escalating to 1000 mg BID as needed and tolerated. Of note, utilizing extended-release metformin may reduce the risk of metformin-related diarrhea in combination with capivasertib^61,62^.

Assess estimated glomerular filtration rate (eGFR) before initiating metformin, as metformin use is contraindicated for eGFR <30 mL/min/1.73 m^2^ and is not recommended for eGFR between 30–45 mL/min/1.73 m^2^ ^16,63^. In patients with eGFR <30 mL/min/1.73 m^2^, or as indicated per local institutional protocols, withhold metformin prior to contrast imaging and 48 h after imaging to minimize the risk of acute kidney injury^64^.

In patients presenting with FG between the upper limit of normal and 160 mg/dL (8.9 mmol/L), no capivasertib dose modifications are required. If not already initiated as prophylaxis, begin treatment with extended-release or standard-release metformin 500 mg/day, escalating as outlined above^11,15^. For FG of 161–250 mg/dL (9–13.9 mmol/L), withhold capivasertib until FG decreases to <160 mg/dL, then resume as outlined in Fig. 4. For patients not already receiving metformin, initiate extended-release metformin at 500 mg/day and escalate to 2000 mg QD, or initiate standard-release metformin at 500 mg/day and escalate to 1000 mg BID, as needed and tolerated. If the patient is already taking prophylactic metformin, consider dose escalation and consider endocrinology consultation^15^. If FG remains high despite treatment with metformin or if metformin dose escalation is limited by toxicities, consider adding an additional agent that does not impact the PI3K/AKT pathway, such as a sodium-glucose cotransporter 2 inhibitor (SGLT2i), thiazolidinediones (TZDs), or α-glucosidase inhibitors (see Fig. 4)^15,23,39,65–67^. Monitor for euglycemic DKA (checking urine or blood ketone levels) in patients receiving SGLT2i, and advise urgent care if DKA symptoms appear^65^. Avoid insulin secretagogues such as sulfonylureas, GLP-1R agonists, dipeptidyl peptidase-4 inhibitors, and meglitinides, as they are associated with AEs such as GI AEs (GLP-1R agonists) and hypoglycemia^65,68,69^.

For FG of 251–500 mg/dL (14–27.8 mmol/L), withhold capivasertib until FG decreases to <160 mg/dL, then resume or permanently discontinue as outlined in Fig. 4. Add SGLT2i or TZD to metformin and consult an endocrinologist. For FG > 400 mg/dL (22.2 mmol/L), consider insulin (0.1 μ/kg/h) as clinically indicated for the shortest duration required to normalize blood glucose while optimizing the dose of other agents. For FG > 500 mg/dL (27.8 mmol/L) or life-threatening complications of hyperglycemia at any FG level, withhold capivasertib and initiate aggressive hydration, electrolyte management, and insulin (0.1 μ/kg/h), as needed^11,39^. Monitor for DKA by measuring serum bicarbonate, electrolytes, ketones, anion gap, and white blood cell count as clinically appropriate^70^. Capivasertib should be permanently discontinued for confirmed DKA, or if FG persists at ≥500 mg/dL for ≥24 h^11^.

## Conclusions

Capivasertib, in combination with fulvestrant, is approved in the US for the treatment of HR-positive/HER2-negative locally advanced or metastatic BC with ≥1 *PIK3CA*, *AKT1*, and/or *PTEN* alterations, following disease progression on ≥1 ET-based regimen in the metastatic setting or recurrence on or within 12 months of completing adjuvant endocrine. The most frequent AEs associated with capivasertib (diarrhea, rash, and hyperglycemia) can impact treatment adherence and persistence, leading to dose modifications. To maximize therapeutic potential, we recommend a multifaceted approach to AE prevention, monitoring, and management. This approach includes patient education, prophylactic measures to prevent rash and hyperglycemia, AE risk assessment and monitoring, and prompt supportive care by all members of the healthcare team. A comprehensive and structured strategy for managing AEs commonly associated with capivasertib can be implemented using the practical recommendations provided here to improve patients’ experience and optimize therapeutic benefit.

## Data Availability

Data sharing is not applicable to this article, as no datasets were formally reviewed in the course of writing this review article.
